# The Smoothing Artifact of Spatially Constrained Canonical Correlation Analysis in Functional MRI

**DOI:** 10.1155/2012/738283

**Published:** 2012-12-24

**Authors:** Dietmar Cordes, Mingwu Jin, Tim Curran, Rajesh Nandy

**Affiliations:** ^1^Department of Physics, Ryerson University, Toronto, ON, Canada M5B 2K3; ^2^Department of Psychology and Neuroscience, University of Colorado, Boulder, CO 80309, USA; ^3^Department of Radiology, University of Colorado, Denver, CO 80045, USA; ^4^Department of Physics, University of Texas, Arlington, TX 76019, USA; ^5^Departments of Biostatistics and Psychology, UCLA, Los Angeles, CA 90095, USA

## Abstract

A wide range of studies show the capacity of multivariate statistical methods for fMRI to improve mapping of brain activations in a noisy environment. An advanced method uses local canonical correlation analysis (CCA) to encompass a group of neighboring voxels instead of looking at the single voxel time course. The value of a suitable test statistic is used as a measure of activation. It is customary to assign the value to the center voxel; however, this is a choice of convenience and without constraints introduces artifacts, especially in regions of strong localized activation. To compensate for these deficiencies, different spatial constraints in CCA have been introduced to enforce dominance of the center voxel. However, even if the dominance condition for the center voxel is satisfied, constrained CCA can still lead to a smoothing artifact, often called the “bleeding artifact of CCA”, in fMRI activation patterns. In this paper a new method is introduced to measure and correct for the smoothing artifact for constrained CCA methods. It is shown that constrained CCA methods corrected for the smoothing artifact lead to more plausible activation patterns in fMRI as shown using data from a motor task and a memory task.

## 1. Introduction

Local canonical correlation analysis (CCA) is a multivariate statistical method in fMRI that uses the joint time course of a group of neighboring voxels, usually in a 3 × 3 in-plane voxel grid, to determine the significance of activation. The value of a suitable test statistic is used as a measure of activation. Since the joint time course of the neighborhood is used, it is not immediately clear to which voxel the measure of activation should be assigned. For example, if a 3 × 3 voxel neighborhood is chosen and the measure of activation is significant, without further assumptions one can only conclude that activation occurred somewhere within the 3 × 3 voxel neighborhood. If the activation is assigned to all voxels of the neighborhood, loss of spatial specificity will occur. To increase spatial specificity, it has been proposed to assign the measure of activation to the center voxel of the 3 × 3 neighborhood [1, 2]. A center voxel assignment is usually justified by mathematical convenience but can also be reasoned on the fact that the fMRI BOLD response leads to patches of activation patterns that are most likely of convex shape and simple connectivity (without any holes in the interior neighborhood). However, this center voxel assignment proved to be prone to yield artifacts as activations tend to bleed to the neighboring voxels of strongly active voxels. The result is a loss of spatial specificity from this smoothing artifact. 

The smoothing artifact is not only common in conventional CCA, but also in any analysis technique that involves spatial low-pass filter kernels, such as univariate (single voxel) analysis where the data have been preprocessed using Gaussian spatial smoothing. In conventional data smoothing, the smoothing artifact has been intentionally “induced” to increase the signal-to-noise ratio at the cost of reduced specificity and occurrence of typical spatial low-pass artifacts such as blurring of edges of activation patterns. 

To compensate for the smoothing artifact in conventional CCA, different assignment schemes were proposed. For example, a minimum relative weight for the center voxel was used to restrict false activations [3]. In another study using a more adaptive approach, the smoothing artifact was reduced by utilizing the spatial dependence among voxels as much as possible and assigning the significance of activation to the dominant voxel of local maxima [4]. This method was shown to be effective in eliminating the smoothing artifact in motor activation data that is known to have large contrast-to-noise ratio (CNR), however, in data where the activation is more subtle (such as hippocampal activation using an episodic memory paradigm), the method has the disadvantage of being less sensitive, according to our studies. 

To reduce the smoothing artifact in CCA, it is necessary to constrain the spatial weights properly and impose the condition that the center voxel always has the largest weight. Constrained CCA (cCCA) with positivity constraints have been proposed for fMRI. Friman et al. [5] as well as Ragnehed et al. [6] use nonnegative spatial weights with maximum weight of the center voxel in order to ensure spatial low-pass filter properties of cCCA. This has the additional benefit of constraining CCA to eliminate spurious correlations occurring in conventional CCA where spatial filters can have positive and negative coefficients.

To our knowledge, the smoothing artifact in cCCA has never been studied. Recently, we provided a mathematical framework for cCCA and computed ROC properties of cCCA with different linear constraints and a nonlinear constraint for activation patterns of motor data and episodic memory data [7, 8]. In this paper we expand our previous research and investigate in detail the smoothing artifact that is associated with each spatial constraint in cCCA. Furthermore, we provide a novel approach of how to correct the measure of activation for the smoothing artifact. Results for motor activation data and episodic memory activation data are presented. Parts of this paper have been published in abstract form (one page) at a recent conference [9].

## 2. Theory

### 2.1. Constrained CCA (cCCA)

In the following we briefly review CCA and cCCA, and explicitly consider the constraints introduced recently [8]. Mathematically, CCA is a generalization of the General Linear Model (GLM) by allowing the incorporation of spatial basis functions according to

(1) (α1f1(ξ)+⋯+αsfs(ξ))⊗Y(ξ,t)  =β1x1(t)+⋯+βrxr(t)+ε(t),

where the data are given by *Y*(*ξ*, *t*), *ξ* is the vector representing the spatial coordinates *x*, *y*, and *z*, and *t* is time. The functions *f*_*i*_(*ξ*), *i* = 1,…, *s* represent the spatial basis functions modeling the activation pattern in a neighborhood. The functions *x*_*j*_(*t*), *j* = 1,…, *r* are the temporal basis functions modeling the signal observed (which is the result of a convolution of the hemodynamic response function and the stimulus function). The coefficients *α*_*i*_ and *β*_*j*_ are the spatial and temporal weights, respectively, that are being determined and optimized by the data for each individual neighborhood using an optimization routine. The symbol ⊗ denotes spatial convolution and *ε*(*t*) is a Gaussian-distributed random error term. If the number of spatial basis functions is reduced to a single function, (1) becomes

(2) $$\begin{array}{c} f_{1}\left(\xi \right)⊗Y\left(\xi ,t\right)=\beta_{1}x_{1}\left(t\right)+\cdots +\beta_{r}x_{r}\left(t\right)+\varepsilon \left(t\right). \end{array}$$

When *f*_1_(*ξ*) is a simple Gaussian function, we obtain the conventional GLM used frequently in fMRI.

Equation (1) can be represented conveniently in matrix form. In the following we assume that the functions *f*_*i*_(*ξ*), *i* = 1,…, *s* are spatial Dirac delta functions defining a local neighborhood within a 3 × 3 pixel neighborhood (*s* ≤ 9). Let **Y** be the matrix representing *s* voxel time courses with dimension *t* × *s* and **X** the conventional design matrix of size *t* × *r* for the *r* temporal regressors. Furthermore, let **α** and **β** be two unknown vectors of size *s* × 1 and *r* × 1, respectively. In CCA, we look for the linear combinations of voxel time courses **Y** **α** and temporal regressors **X** **β** such that the correlation between both quantities is maximum. This leads to an eigenvalue problem with min(*s*, *r*) solutions from which the solution with the largest eigenvalue (i.e., maximum canonical correlation) is being chosen. Without constraints on the *α*_*i*_, the specificity of the activation pattern obtained by CCA is low and could result in artifacts (see e.g., [8]). To put constraints on the spatial weights **α** in order to restrict the space of unreasonable solutions for fMRI, we consider the following four scenarios for the components *α*_*i*_ of **α**, where *α*_1_ is the weight for the center voxel and the other *α*_*i*_'s represent the weights for the *s* neighborhood voxels.

 Constraint 1 (Simple Constraint). One has
  (3) $$\begin{array}{c} \alpha_{1}>0,\;\;\alpha_{i}\geq 0\;\forall i\geq 2. \end{array}$$
 Constraint 2 (Sum Constraint). One has
  (4) $$\begin{array}{c} \alpha_{1}\geq \sum_{i=2}^{s}\alpha_{i}>0,\;\alpha_{i}\geq 0\;\forall i\geq 2. \end{array}$$
 Constraint 3 (Average Constraint). One has
  (5) $$\begin{array}{c} \alpha_{1}\geq \frac{1}{s-1}\sum_{i=2}^{s}\alpha_{i}>0,\;\alpha_{i}\geq 0\;\forall i\geq 2. \end{array}$$
 Constraint 4 (Maximum Constraint). One has
  (6) $$\begin{array}{c} \alpha_{1}\geq \max\left(\alpha_{i}\right)>0,\;\alpha_{i}\geq 0\;\forall i\geq 2. \end{array}$$

Note that the neighborhood size *s* is not a fixed quantity, but is determined from the data by cCCA and can differ for each center voxel. 

### 2.2. Smoothing Artifact

The smoothing artifact in CCA is defined as the probability of incorrectly declaring the center voxel of a configuration of size *s* (*s* ≤ 9 for a 3 × 3 neighborhood) to be active. In the following, we outline how to compute the posterior probability to detect the smoothing artifact in real data using a Bayesian framework. The posterior probability, *P*, that a center voxel is not active when it was in fact declared active, is given by

(7) $$\begin{array}{c} P=p\left(\text{center}\;\text{voxel}\;\text{is}\;\text{not}\;\text{active}\;|\;\omega >\omega_{0},\text{cnr},M,\text{CNR},s\right), \end{array}$$

where *ω* > *ω*_0_ indicates that the center voxel was declared active (statistic *ω* > threshold *ω*_0_ with *ω* ∈ [0, *∞*)), cnr is the univariate contrast-to-noise ratio of the center voxel, *M* labels the method of data analysis, CNR is the contrast-to-noise ratio of the entire configuration defining the neighborhood within a 3 × 3 pixel region, and *s* is the size of the configuration (i.e., number of declared active voxels ≤9 within the neighborhood). For abbreviation, we define the set of parameters, *θ*, to be

(8) $$\begin{array}{c} \theta =\left\{\text{cnr},M,\text{CNR},s\right\}. \end{array}$$

Then, according to Bayes' theorem for conditional probabilities, (7) can be written as

(9) P=p(ω>ω0 ∣ center  voxel  is  not  active,θ)×p(center  voxel  is  not  active ∣ θ)(p(ω>ω0 ∣ θ))−1,

which is of the form

(10) $$\begin{array}{c} P=\frac{P_{1}P_{3}}{P_{2}}, \end{array}$$

where

(11) $$\begin{array}{c} P_{1}=p\left(\omega >\omega_{0}\;|\;\text{center}\;\text{voxel}\;\text{is}\;\text{not}\;\text{active},\theta \right), \end{array}$$

(12) $$\begin{array}{c} P_{2}=p\left(\omega >\omega_{0}\;|\;\theta \right), \end{array}$$

(13) $$\begin{array}{c} P_{3}=p\left(\text{center}\;\text{voxel}\;\text{is}\;\text{not}\;\text{active}\;|\;\theta \right). \end{array}$$

The term *P*_1_ is called the bleeding artifact because it represents the probability that an inactive voxel is declared as active. We determine *P*_1_ as a function of the size, *s*, of the configuration only and not as a function of the geometrical shape of the configuration. Note that the dependence on *s* is an approximation, because in reality there are 2^8^ = 256 possible configurations that can contain 0 to 8 active voxels (corresponding to *s* ∈ {1,…, 9} since *s* labels the neighborhood size within a 3 × 3 pixel grid, which always includes the center voxel, independent if the center voxel is active or not). Each configuration of size *s* has, depending on its distance of all voxel members to the center voxel, a slightly different value for *P*_1_. For example, configurations with *s* = 7 leads to 3 different classes based on a distance measure, that is, class 1 = {center voxel, 4 corner voxels, and 2 midedge voxels}, class 2 = {center voxel, 3 corner voxels, and 3 midedge voxels}, class 3 = {center voxel, 2 corner voxels, and 4 midedge voxels}. 

According to our simulations, *P*_1_ is strongly dependent on *s* but not on a particular configuration of *s*. Only a weak dependence based on different class memberships exist, which we neglect for the purpose of this research. To estimate *P*_1_, it is thus reasonable to group all configurations for a particular *s* together and compute an average value of *P*_1_ over all possible configurations with size *s*.

#### 2.2.1. Estimation of *P*_1_ (See (11))

The term *P*_1_ can be estimated from simulations using a mixture of resampled resting-state data and activation data at given *θ* using kernel density estimation [10]. Resampled resting-state data are considered null data with respect to any task fMRI function since the temporal structure is destroyed by resampling using the wavelet transform. This resampling, however, does not destroy the autocorrelations inherent in resting-state data. Furthermore, the resampling does not affect the spatial correlations within the data because the permutations of the wavelet coefficients are kept the same for each voxel time series in a particular simulation; however, different simulations use different permutations [11, 12]. 

The simulated data are superpositions of time series from a 3 × 3 pixel neighborhood of null data and activation data. Since the entire neighborhoods are used from resting-state data, realistic spatial correlations of the simulated data are obtained. In particular, for a configuration of *s* active voxels in the 3 × 3 neighborhood, the simulated voxel time courses, *y*_*i*_(*t*), are obtained by

(14) $$\begin{array}{c} y_{i}\left(t\right)=\{\begin{array}{cc} y_{i}^{\left(0\right)}\left(t\right), & \text{for}\;i=1,\\ \beta x\left(t\right)+y_{i}^{\left(0\right)}\left(t\right), & \text{for}\;i\;\in \left\{2,\ldots ,s\right\}, \end{array} \end{array}$$

where *i* = 1 refers to the center voxel and all other *i* to the surrounding voxels of the configuration of size *s* within the 3 × 3 neighborhood. All *y*_*i*_^(0)^(*t*) correspond to resampled resting-state time courses and represent spatially and temporally correlated null (noise) data. Note that the center voxel is always inactive by design to compute *P*_1_. Thus, *P*_1_ is a strong function of CNR of the configuration but not of the value cnr (which is the contrast-to-noise ratio of the inactive center voxel), and the dependence of *P*_1_ on cnr can be neglected. The activation is determined by the hemodynamic response function, *x*(*t*), of interest multiplied by factor *β* so that the configuration has a given CNR. In order to compute the CNR we use the general definition

(15) $$\begin{array}{c} \text{CNR}={\left(\frac{\sum \lambda_{i}}{\sum \varsigma_{i}}\right)}^{1/2}, \end{array}$$

where *λ*_*i*_ and *ς*_*i*_ are the eigenvalues of the covariance matrix of the activation signal and noise, respectively [13]. Note that (15) can be used for a single voxel time series or an entire neighborhood of arbitrary size. To determine the activation signal and noise of a configuration using cCCA, we convert the cCCA problem into a multivariate multiple regression problem of the form

(16) $$\begin{array}{c} Y\alpha =XB\alpha +E\alpha , \end{array}$$

where **Y** are the data (size *t* × *s*), **α** is the optimum spatial weight vector (size *s* × 1), **X** is the design matrix (size *t* × *r*), **B** is the matrix of regression weights (size *r* × *s*), and **E** is a residual error matrix (size *t* × *s*). For a given contrast vector **c**, we reparameterize the design matrix **X** and obtain a transformed design matrix $\tilde{X}$ such that

(17) $$\begin{array}{c} \tilde{X}=\left[X_{\text{eff}\;}X_{⊥}\right], \end{array}$$

where

(18) $$\begin{array}{c} X_{\text{eff}\;}={X\left(X^{\prime }X\right)}^{-1}c{\left(c^{\prime }{\left(X^{\prime }X\right)}^{-1}c\right)}^{-1} \end{array}$$

is the first regressor of the new design matrix $\tilde{X}$ that is associated with a parameter estimate equivalent to the original contrast **c**′**B** **α** [8, 14]. The matrix **X**_⊥_ is perpendicular to **X**_eff_ and plays no role in the estimation of **c**′**β**. Then, the signal **S**(*t*) is obtained by

(19) $$\begin{array}{c} S=X_{\text{eff}}B, \end{array}$$

and the noise **N**(*t*) is obtained by

(20) $$\begin{array}{c} N=\left(Y-X_{\text{eff}}B\right)\alpha . \end{array}$$

#### 2.2.2. Estimation of *P*_2_ (See (12))

This term can be estimated directly from the real data. In this case, for each *M* and *s* > 1, *P*_2_ is a 2D function of cnr and CNR, but depends strongly only on CNR so that the dependence on cnr can be neglected. Note that for *s* = 1, cnr = CNR, and in this case *P*_2_ is a 1D function of cnr only. It is possible to determine first the joint probability density *p*(*ω*, CNR | *s*, *M*) using 2D kernel density estimation with a 2D Gaussian kernel, which then can be integrated numerically to obtain *P*_2_ according to

(21) $$\begin{array}{c} P_{2}\left(\omega_{0},\text{CNR},s,M\right)=\frac{\int_{\omega_{0}}^{\infty }p\left(\omega ,\text{CNR}\;|\;s,M\right)d\omega }{\int_{0}^{\infty }p\left(\omega ,\text{CNR}\;|\;s,M\right)d\omega }. \end{array}$$

Note that *P*_2_(*ω*_0_, CNR, *s*, *M*) for fixed {*ω*_0_, *s*, *M*} has a sigmoidal shape approaching the value 1 for CNR > 0.6. Thus, voxels that are declared active at a family-wise error rate (FWE) <0.05 have necessarily a large CNR for which *P*_2_(*ω*_0_, CNR, *s*, *M*) → 1 (see Section 4).

#### 2.2.3. Estimation of *P*_3_ (See (13))

The term *P*_3_ is less difficult to determine because it is independent of the value of the statistic *ω* and depends strongly on the univariate cnr of the center voxel (configuration with size *s* = 1, and *M* = 1), that is,

(22) P3=p(center  voxel  is  not  active ∣ θ)≈p(center  voxel  is  not  active ∣ cnr,s=1,M=1)=p(center  voxel  is  not  active,cnr ∣ s=1,M=1)p(cnr ∣ s=1,M=1),

where *M* = 1 labels the univariate single voxel analysis method without smoothing. Then, *P*_3_ is only a function of cnr and can be estimated from linear mixture modeling of the real data assuming that the data consists only of active and inactive voxels with unknown fractions. With this assumption, we define the cnr distribution of the data as *h*(cnr), consisting of the mixtures *f*(cnr) and *G*_*μ*,*σ*_(cnr) using

(23) $$\begin{array}{c} h\left(x\right)=a\frac{1}{d}f\left(\frac{\text{cnr}}{d}\right)+\left(1-a\right)G_{\mu ,\sigma }\left(\text{cnr}\right). \end{array}$$

The distribution *f*(cnr) is estimated from resampled resting-state data and the scaled distribution (1/*d*)*f*(cnr/*d*) reflects the null distribution in activation data. The fact that *f*(cnr) is scaled by constant *d* is rooted in the observation that in activation data more neural activity exists and maybe by spatial correlations or other hemodynamic means the distribution of the signal corresponding to inactive voxels is shifted to slightly larger values of cnr. The second term on the right in (23), *G*_*μ*,*σ*_(cnr), represents the cnr distribution of active voxels modeled by a Gaussian distribution with mean *μ* and variance *σ*. All the parameters *a*, *d*, *μ*, and  *σ* are obtained from least squares fitting using activation data. Then,

(24) $$\begin{array}{c} P_{3}\left(\text{cnr}\right)=\frac{a\left(1/d\right)f\left(\text{cnr}/d\right)\;}{h\left(\text{cnr}\right)}. \end{array}$$

#### 2.2.4. Final Result of Estimation of *P* (See (9) and (10))

Overall, the posterior probability that a center voxel is not active is given by

(25) P=p(center  voxel  is  not  active ∣ ω>ω0,θ)≈p(ω>ω0 ∣ center  voxel  is  not  active,θ)P3(cnr)p(ω>ω0 ∣ θ)≈P1(ω0,CNR,M,s)P3(cnr),

since *p*(*ω* > *ω*_0_ | *θ*) → 1 for voxels declared highly active (i.e., FWE <0.05). In the following, we call the function *P*_1_(*ω*_0_, CNR, *M*, *s*) the smoothing artifact function. To correct for the smoothing artifact we propose the rule:

(26) $$\begin{array}{c} \text{Voxel}\;\text{is}\;\text{assigned}\;\text{to}\;\text{be}\;\text{inactive}\;\text{if}\;P>0.5 \end{array}$$

and assign zero to the measure of activation if this statement is true. If this statement is not true, the measure of activation is unchanged.

## 3. Materials and Methods

 FMRI was performed for 6 normal subjects with IRB approval (according to institutional requirements) in a 3.0T GE HDx MRI scanner equipped with an 8-channel head coil and parallel imaging acquisition using EPI with imaging parameters: ASSET = 2, ramp sampling, TR/TE = 2 sec/30 ms, FA = 70 deg, FOV = 22 cm × 22 cm, thickness/gap = 4 mm/1 mm, 25 slices, and resolution 96 × 96. Three fMRI data sets were obtained for each subject. In the following we briefly describe the paradigms and refer the reader for more detail to our previous article [7].

 The first data set was collected during resting-state where the subject tried to relax and refrain from executing any overt task with eyes closed. The second data set was collected while the subject was performing an episodic memory task with oblique coronal slices collected perpendicular to the long axis of the hippocampus. Specifically, this task consisted of memorization of novel faces paired with occupations and contained 6 periods of encoding, distraction, and recognition tasks as well as short instructions where words on the screen reminded subjects of the task ahead. The third data set was obtained by performing an event-related motor task involving bilateral finger tapping while the subject was looking at a screen. 

### 3.1. Data Analysis

All fMRI data were realigned using Statistical Parametric Mapping (SPM5, http://www.fil.ion.ucl.ac.uk/spm/) and maximum motion components were found to be less than 0.6 mm in all directions. In a preprocessing step, all voxel time series were corrected for different slice timings and high-pass filtered by regression using a discrete cosine basis with cut-off frequency 1/120 Hz [15]. No temporal low-pass filtering was carried out. All voxels with intensity larger than 10% of the mean intensity were used in the analysis. This threshold effectively eliminated all nonbrain voxels leading to an average of about 4500 voxels per slice. All activation maps were thresholded using a FWE <0.05 determined by using nonparametric methods [7, 16] with wavelet resampled resting-state data [11, 12].

### 3.2. Basis Functions for CCA

All voxel time courses and temporal regressors were mean subtracted (over time) and variance normalized. As local spatial basis functions we use Dirac delta functions in each 3 × 3 in-slice neighborhood. For the temporal modeling, we specified design matrices as in SPM5 containing all conditions of the paradigms. In particular, for the memory paradigm we modeled instruction (I), encoding (E), recognition (R), and control (C) by temporal reference functions whereas for the motor paradigm, fixation (F), and motor task (M) were modeled according to the paradigm timings. All reference functions were convolved as usual with the standard SPM5 two-gamma hemodynamic response function. For the motor task we computed activation maps for the contrast M-F, and for the memory task we used the contrast E-C. We used reparameterization of the design matrix **X** (see (18)) to incorporate the contrast of interest and optimized the spatial coefficients for each spatial constraint using the methods proposed in our previous publication [8].

## 4. Results and Discussion

### 4.1. The Smoothing Artifact Function

**Figure 1:**
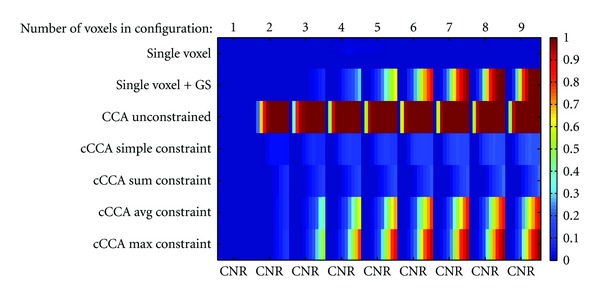
Smoothing artifact of the center voxel for different analysis methods as a function of the configuration size and CNR for simulated motor activation data. Note that the figure is composed of 9 separate images belonging to configuration sizes 1 to 9, and each image for a particular configuration size is a function of the CNR ranging from 0 to 1 in steps of 0.1 in the horizontal direction. The center voxel for each configuration is by design nonactive and the other members (neighboring voxels belonging to the particular configuration) are active with given CNR. In general the smoothing artifact increases with increasing CNR except for single-voxel analysis, where no artifact exists (as expected). Regarding the computation of the false positive fraction, the statistical thresholds were chosen corresponding to a family-wise error rate (FWE) < 0.05 for re-sampled restingstate data.

Using simulated data, the smoothing artifact function *P*_1_ (see (11)) was determined for the motor paradigm with contrast M-F and memory paradigm with contrast E-C, respectively. Simulations were carried out for all methods *M* (single voxel analysis, single voxel analysis with Gaussian spatial smoothing, unconstrained CCA, CCA with the simple constraint, CCA with the sum constraint, CCA with the average constraint, and CCA with the maximum constraint), CNR in the range [0,1] in steps of 0.1, and configuration sizes 1 to 9. All possible 256 configurations in a 3 × 3 neighborhood with inactive center voxel were simulated 1000 times and then regrouped according to the sizes *s* = {1,…, 9}.  Figure 1 shows the smoothing artifact function for the motor paradigm for a typical subject. An almost identical figure was obtained for the memory paradigm. The threshold *ω*_0_ corresponds to FWE = 0.05. Please note that this figure is a composition of nine different images where each image belongs to a configuration of a particular size (1 to 9) and each abscissa is the CNR ranging from 0 to 1 in steps of 0.1. The vertical axis labels the different analysis methods applied and the color determines the value of *P*_1_, ranging from 0 to 1. Bluish color indicates that the smoothing artifact is negligible whereas red color indicates that the smoothing artifact is significant. It is obvious that single-voxel analysis without Gaussian smoothing does not show any smoothing artifact and single-voxel analysis with spatial smoothing leads to a significant smoothing artifact, the larger the CNR and the larger the neighborhood is. It is also obvious that unconstrained CCA has the largest smoothing artifact and this artifact is already large for configuration sizes of *s* = 2 and CNR = 0.2. However, choosing the simple nonnegativity constraint for cCCA almost completely eliminates the smoothing artifact (*P*_1_ < 0.35). Similarly, cCCA with the sum constraint has a smoothing artifact that is even lower (*P*_1_ < 0.3) and should be considered the method of choice if a high specificity is desirable. The cCCA methods with the more complicated constraints (avg constraint and max constraint) show a significant smoothing artifact for configuration sizes of *s* ≥ 3, as long as the CNR is large (CNR > 0.6). These two cCCA methods have very high sensitivity but can lead to false activations when the configuration size is large.

### 4.2. Density Estimation of *P*_2_ (See (21))

**Figure 2:**
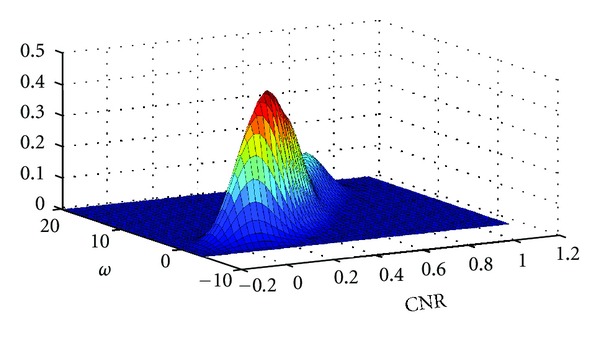
Example of the calculated joint probability density *p*(*ω*, CNR | *s*, *M*) for motor activation data for a neighborhood size *s* = 5 and cCCA with the maximum constraint. The function was determined using 2D kernel density estimation with a 2D Gaussian kernel. The variables *ω* and CNR specify the statistic and the contrast-to-noise ratio of the 5-voxel neighborhood, respectively. The threshold to obtain FWE <0.05 is *ω*_0_ = 6.7.

**Figure 3:**
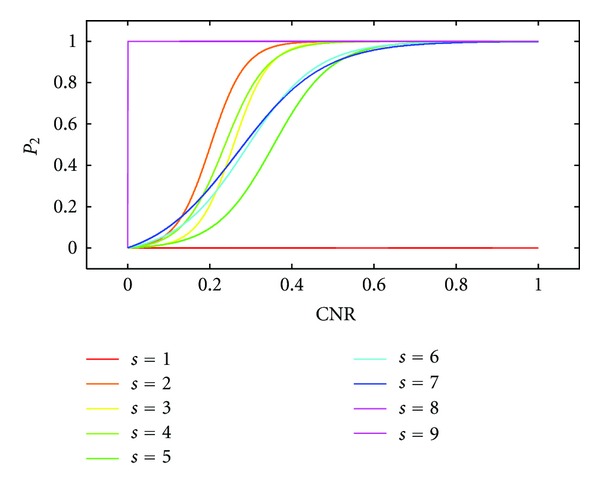
Estimation of *P*_2_(*ω*_0_, CNR, *s*, *M*) = ∫_*ω*_0__^*∞*^ *p*(*ω*, CNR | *s*, *M*)*dω*/∫_0_^*∞*^ *p*(*ω*, CNR | *s*, *M*)*dω* from motor activation data using numerical integration for all configurations of size *s* ∈ {1,…, 9} for cCCA with the maximum constraint. The joint probability density *p*(*ω*, CNR | *s*, *M*) in the integrands are determined with 2D kernel density estimation using a 2D Gaussian kernel (for an example see Figure 2). Note that for CNR > 0.6, *P*_2_ approaches the value 1 rapidly for all *s* ≠ 1. This relationship is also true for data obtained from the memory experiment. Note that configurations with *s* = 1 have no significance in contributing to the smoothing artifact because the smoothing artifact is by definition equal to zero for *s* = 1.

The function *P*_2_(*ω*_0_, CNR, *s*, *M*) = ∫_*ω*_0__^*∞*^ *p*(*ω*, CNR | *s*, *M*)*dω*/∫_0_^*∞*^ *p*(*ω*, CNR | *s*, *M*)*dω* was calculated in MATLAB (http://www.mathworks.com/) by 2D kernel density estimation of *p*(*ω*, CNR | *s*, *M*) using an optimum bandwidth estimator according to Sheather and Jones [17]. In general, *p*(*ω*, CNR | *s*, *M*) has a bimodal distribution for configuration sizes *s* ∈ {2,3, 4,5, 6,7}. For lower *s*, the larger mode of the density occurs at lower values of {*ω*, CNR}, whereas for larger values of *s*, the larger mode occurs at higher values of {*ω*, CNR}. For *s* ∈ {8,9}, the density becomes unimodal with mode located at large values of {*ω*, CNR}. Also note, that *ω* is strongly correlated with *CNR*, which is expected. An example of *p*(*ω*, CNR | *s*, *M*) is given in Figure 2 for *s* = 5 and cCCA with the maximum constraint. The shape of *P*_2_(*ω*_0_, CNR, *s*, *M*) obtained by numerical integration of ∫_*ω*_0__^*∞*^ *p*(*ω*, CNR | *s*, *M*)*dω*/∫_0_^*∞*^ *p*(*ω*, CNR | *s*, *M*)*dω* and density smoothing is shown in Figure 3 for all *s* and 0 ≤ CNR ≤ 1. Note the S-shaped form obtained for *P*_2_(*ω*_0_, CNR, *s*, *M*) for all integrations of bimodal distributions involving *p*(*ω*, CNR | *s*, *M*), whereas for *s* = 1 the function *P*_2_ is zero for CNR ≤ 1 and for *s* ∈ {8,9}  *P*_2_ has the value 1 for 0 < CNR ≤ 1. The function *P*_2_ for size *s* = 1 plays no role in determining the posterior probability *P* because the smoothing artifact is zero by definition, since a single-voxel-neighborhood cannot have any bleeding of signal strength. 

### 4.3. Density Estimation of the Null cnr Distribution in Activation Data

**Figure 4:**
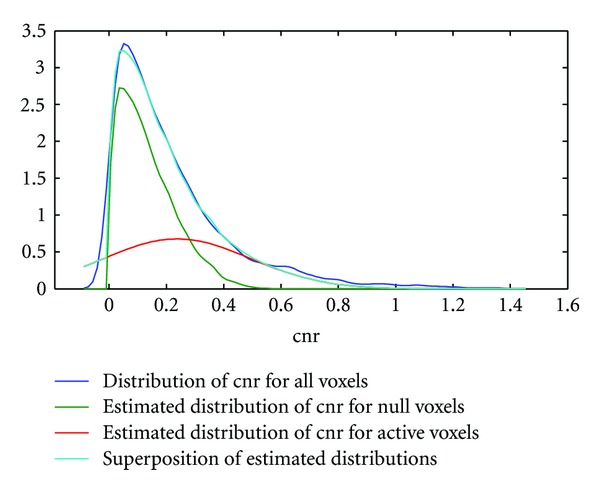
Density estimation of active and inactive voxels in motor activation data. The top curve (dark blue) shows the distribution of the cnr using kernel density estimation techniques. The green and red curves are the estimated distributions of the cnr for the inactive and active voxels, respectively. Note that the distribution of the inactive voxels (green curve) was derived from wavelet resampled resting-state data using a dilation variable, whereas the distribution of the active voxels (red curve) was derived from a Gaussian distribution. The mixture of the estimated distributions for null and active voxels is given by the light blue curve showing very small differences to the raw curve (dark blue).

**Figure 5:**
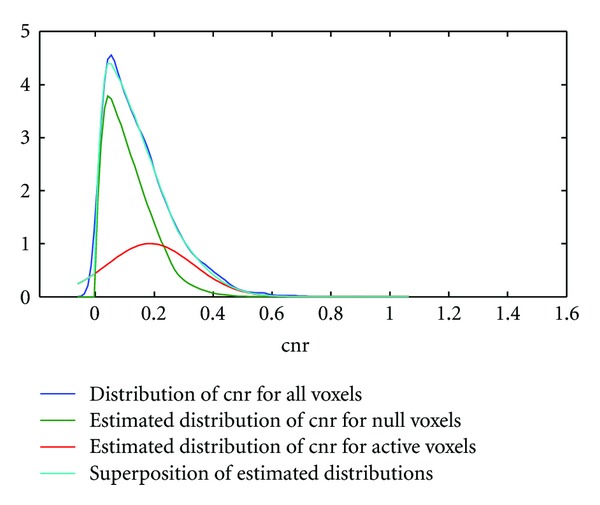
Density estimation of active and inactive voxels in memory activation data. The top curve (dark blue) shows the distribution of the cnr using kernel density estimation techniques. The green and red curves are the estimated distributions of the cnr for the inactive and active voxels, respectively. Note that the distribution of the inactive voxels (green curve) was derived from wavelet resampled resting-state data using a dilation variable, whereas the distribution of the active voxels (red curve) was derived from a Gaussian distribution. The mixture of the estimated distributions for null and active voxels is given by the light blue curve showing very small differences to the raw curve (dark blue).

In Figure 4 we computed the null cnr density function (1/*d*)*f*(cnr/*d*) using real motor activation data of a typical subject and obtained a dilation parameter *d*  =  1.26, indicating that the null distribution of the cnr obtained from resampled resting-state data is slightly inflated in activation data. The overall fit of the density functions (1/*d*)*f*(cnr/*d*) and *G*_*μ*,*σ*_(cnr) is good leading to a small residual mean squared error = 0.014 ± 0.114 (compare the light blue curve and the dark blue curve in Figure 4). A very similar curve was obtained for the memory paradigm using data from a different subject. Here the dilation parameter was found to be *d* = 1.27 and mean squared error = 0.011 ± 0.105 (Figure 5). Overall, the density fits obtained were similar.

### 4.4. Correcting the Smoothing Artifact in Motor and Memory Data

**Figure 6:**
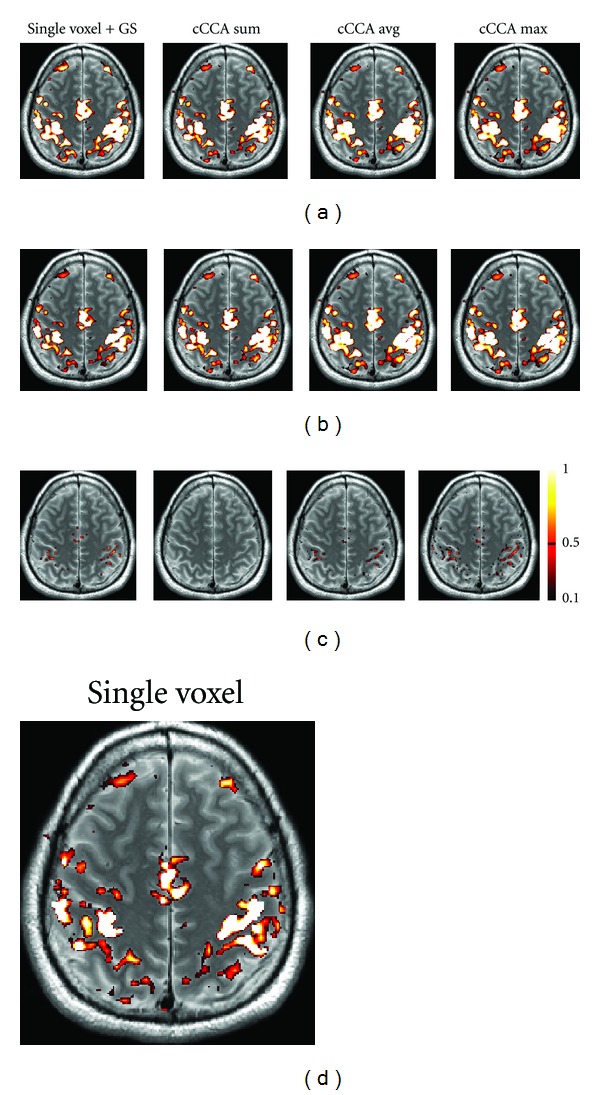
Motor activation maps for contrast “motor” minus “fixation” using different data analysis methods (single voxel with Gaussian smoothing (GS), CCA with the sum constraint (cCCA sum), CCA with the average constraint (cCCA avg), CCA with the maximum constraint (cCCA max)). In (a), original activations maps are shown at FWE <0.05. In (b), activation maps corrected for the smoothing artifact are shown. Corrections are done for *P* > 0.5. In (c), voxels affected by the smoothing artifact are shown. The color scale on the right refers to the magnitude of the smoothing artifact in (c). In (d) we show for comparison the activation map for single voxel analysis without Gaussian smoothing.

**Figure 7:**
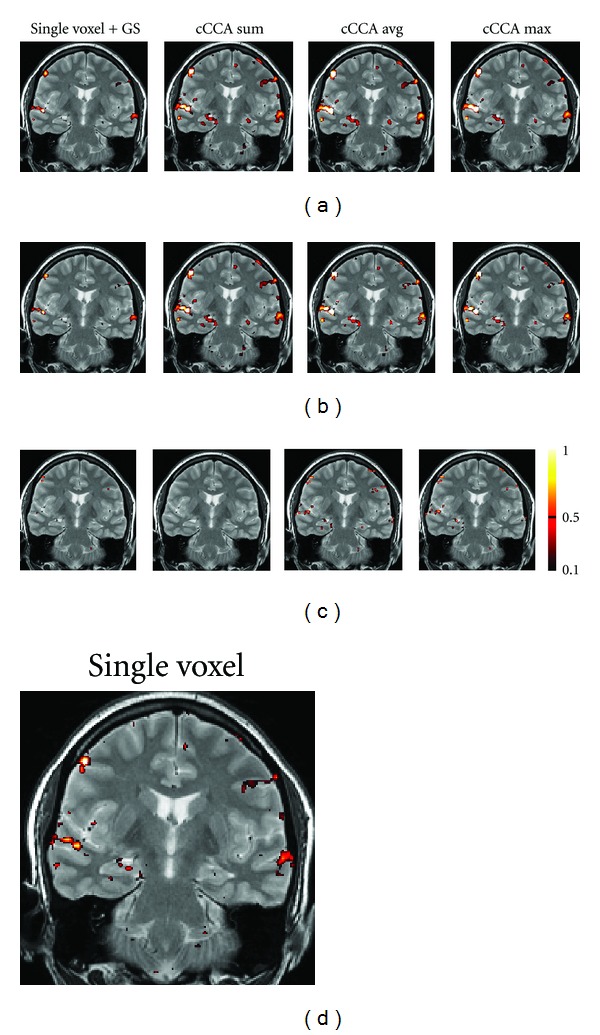
Memory activation maps for contrast “encoding” minus “control” using different data analysis methods (single voxel with Gaussian smoothing (GS), CCA with the sum constraint (cCCA sum), CCA with the average constraint (cCCA avg), and CCA with the maximum constraint (cCCA max)). In (a), original activations maps are shown at FWE <0.05. In (b), activation maps corrected for the smoothing artifact are shown. Corrections are done for *P* > 0.5. In (c), voxels affected by the smoothing artifact are shown. The color scale on the right refers to the magnitude of the smoothing artifact in (c). In (d) we show for comparison the activation map for single-voxel analysis without Gaussian smoothing.

**Figure 8:**
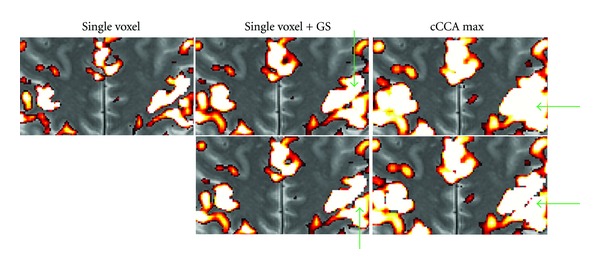
Magnified motor activation maps from Figure 6 for selected analysis methods (single voxel without Gaussian smoothing, single voxel with Gaussian smoothing (GS), and CCA with the maximum constraint (cCCA max)). The top row shows the original images at FWE <0.05 without any correction for the smoothing artifact. The bottom row shows the original images corrected for the smoothing artifact. The green arrows point to major differences of the activation patterns. Note that correction for the smoothing artifact leads to a splitting of activation pattern in the left (radiological convention) motor cortex (see bottom row images with green arrows pointing to the ROI).

**Figure 9:**
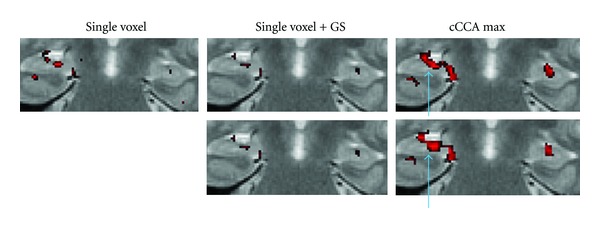
Magnified memory activation maps from Figure 7 for selected analysis methods (single voxel without Gaussian smoothing, single voxel with Gaussian smoothing (GS), and CCA with the maximum constraint (cCCA max)). The top row shows the original images at FWE <0.05 without any correction for the smoothing artifact. The bottom row shows the original images corrected for the smoothing artifact. The blue arrows point to major differences of the activation patterns. Note that hippocampal activation after correction for the smoothing artifact appears to have three separate foci in the right temporal lobe (radiological convention) as shown in the bottom image on the right (blue arrow).

In Figures 6 and 7 we show examples of the severity of the smoothing artifact for activation data thresholded at the *P* < 0.05 level, corrected for multiple comparison (i.e., FWE <0.05). The number of voxels affected by the smoothing artifact can be considerable for single voxel with Gaussian smoothing and cCCA with the average constraint as well as cCCA with the max constraint, as seen in motor data (Figure 6). For cCCA with the sum constraint, however, there is no correction for the smoothing artifact necessary because the sum constraint produces a sufficiently dominant weight for the center voxel so that inactive voxels cannot obtain a dominant weight in the neighborhood of active voxels. We did not find any voxel with a smoothing artifact >0.1 confirming that cCCA with the sum constraint has largest specificity of the proposed cCCA methods. The activation patterns that are corrected for the smoothing artifact show small changes compared to the uncorrected ones, however, these changes can provide important information of the activation profile. For example, in Figures 8 and 9 we show a magnified region of the left motor cortex and the right hippocampus, respectively, for selected analysis methods (single voxel with and without Gaussian smoothing, cCCA with the maximum constraint). Here we see that correction for the smoothing artifact leads to a separation of the right motor cortex (see green arrows in Figure 8). This result is consistent with the activation pattern from single voxel analysis without Gaussian smoothing. We believe that for the motor activation data, single-voxel analysis is already accurate due to the high cnr of the BOLD response for motor activation. Regarding the hippocampal activation, we see that the correction for the smoothing artifact leads to a clear separation of hippocampal activation into three focal regions (see blue arrow in Figure 9). It is conceivable that the corrected activation maps are more accurate representations of true hippocampal activations in this high-resolution study because it is known that the hippocampus is composed of the CA fields (CA1, CA2, CA3, and CA4), the dentate gyrus and subiculum, and each of these subregions has a specific function in memory. The obtained corrections of the activation pattern are more probable than a continuous elongated activation pattern obtained with cCCA without correction for the smoothing artifact. 

**Figure 10:**
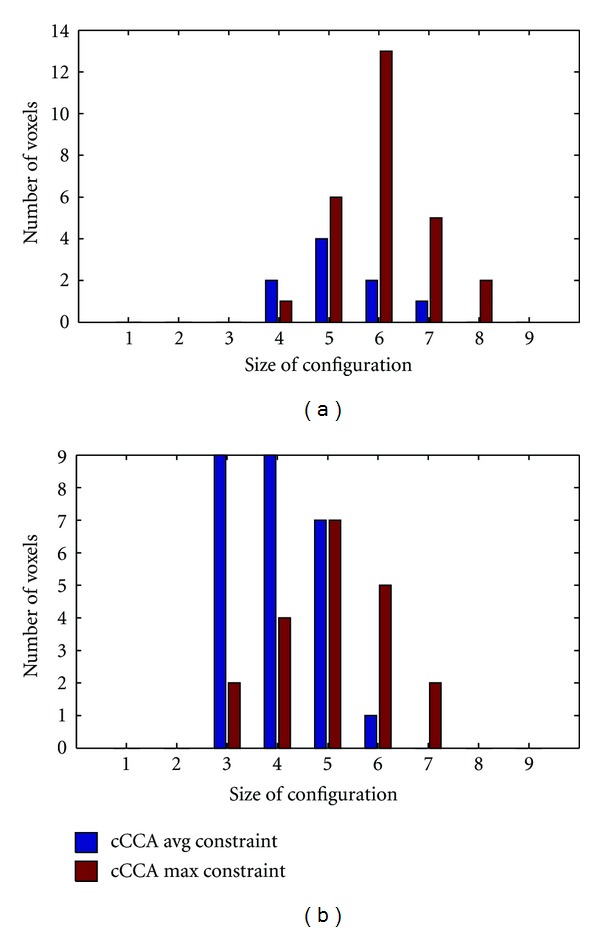
Number of voxels affected by the smoothing artifact for two different constrained CCA methods (CCA with the average constraint (cCCA avg) and CCA with the maximum constraint (cCCA max)) as a function of the neighborhood size of the configuration. (a) is for motor data, (b) for memory data (obtained from Figures 6 and 7, resp.). Note that CCA with the max constraint leads to configurations of larger neighborhoods that need to be corrected for the smoothing artifact compared to CCA with the average constraint. The result for CCA with the sum constraint is not shown because the smoothing artifact associated with this constraint is negligible (compare Tables 1 and 2).

We chose to correct the smoothing artifact when *P* > 0.5. This condition is still a conservative correction for activation maps. To obtain better specificity but at a cost of losing sensitivity, it may be worthwhile to lower the threshold. Tables 1 and 2 show the number of voxels affected by the smoothing artifact for thresholds 0.1 to 0.5. Note that lowering the threshold for *P* to 0.2 leads to a dramatic increase in the number of voxels. Thus, *P* > 0.2 should be avoided. The choice *P* > 0.3 is probably a good compromise of achieving better specificity and still maintaining high sensitivity for the examples shown here. However, the decision to use a lower threshold than 0.5 will primarily dependent on the particular application of the research. We preferred *P* > 0.5 which lead to a relatively small number of voxels that needed to be corrected. With this threshold the sensitivity of the methods is still very large and mostly voxel configurations of sizes 4 to 8 in motor data and 3 to 7 in memory data were affected by the smoothing artifact (Figure 10). Note that cCCA with the max constraint leads to larger configuration sizes (mean value *s* = 5.6) that are affected by the smoothing artifact than cCCA with the average constraint (mean value *s* = 4.3). This fact is expected due to the increased freedom of the spatial constraints in cCCA with the maximum constraint leading, on average, to larger configuration sizes which are more probable to induce a smoothing artifact than the other constrained cCCA methods. 

## 5. Conclusions

 We summarize the ideas introduced in this study and results obtained as follows.

1. We investigated the smoothing artifact in CCA and proposed a new technique to reduce this artifact in fMRI data analysis.
2. Using data from a motor activation paradigm and an episodic memory paradigm, we showed examples of activation maps obtained with constrained CCA methods, the corresponding magnitude of the smoothing artifact, and activation maps corrected for the smoothing artifact.
3. For all data studied, we found no appreciable smoothing artifact for cCCA with the sum constraint.
4. The best overall performance was obtained by cCCA with the maximum constraint corrected for the smoothing artifact. We recommend this technique for fMRI data analysis to obtain high sensitivity and good specificity.

## Figures and Tables

**Table 1:** Number of voxels affected by the smoothing artifact for different data analysis methods as a function of *P*> threshold in motor activation data for contrast “motor” minus “fixation”.

Threshold	SV + GS	cCCA sum	cCCA avg	cCCA max
0.5	34	0	9	27
0.4	38	0	14	40
0.3	40	0	24	51
0.2	42	0	48	104
0.1	43	0	98	174

**Table 2:** Number of voxels affected by the smoothing artifact for different data analysis methods as a function of *P*> threshold in memory activation data for contrast “encoding” minus “control”.

Threshold	SV + GS	cCCA sum	cCCA avg	cCCA max
0.5	5	0	26	20
0.4	5	0	32	20
0.3	6	0	37	24
0.2	7	0	45	33
0.1	10	0	55	44
